# Using health belief model constructs to understand the role of perceived disease threat and resilience in responding to COVID-19 among people who use drugs: a cluster analysis

**DOI:** 10.3389/adar.2024.12197

**Published:** 2024-07-08

**Authors:** Kirsten Paulus, Sarah Bauerle Bass, Patrick J. A. Kelly, Jenine Pilla, AnnaMarie Otor, Madison Scialanca, Anamarys Arroyo, Namaijah Faison

**Affiliations:** ^1^ Department of Social and Behavioral Sciences, Temple University College of Public Health, Philadelphia, PA, United States; ^2^ Risk Communication Laboratory, Temple University College of Public Health, Philadelphia, PA, United States

**Keywords:** people who use drugs, health belief model, COVID-19, infectious disease, resilience

## Abstract

**Introduction:**

The Health Belief Model (HBM) has been successfully applied to understanding adherence to COVID-19 prevention practices. It has not, however, been used to understand behavior in people who use drugs (PWUD). The aim of this study was to use the HBM to better understand COVID-19 perceptions among PWUD and understand how resiliency affects those perceptions.

**Materials and methods:**

A cross-sectional survey was completed from September to December 2021 with PWUD (n = 75) who utilize services at a large harm reduction organization in Philadelphia. Segmentation analysis was done using a k-means clustering approach. Two clusters emerged based on perceived COVID-19 personal impact and resiliency (Less COVID impact/High resilience (NoCOV/HR) and High COVID impact/Low resilience (COV/LR). Differences in responses by cluster to perceptions of COVID-19 and individual pandemic response grouped by HBM constructs were assessed using Student’s t-test and chi squares.

**Results:**

Significant differences in HBM constructs were seen between clusters. Those in the ***COV/LR*** cluster were more likely to think they were susceptible to getting COVID-19 and less likely to believe they knew how to protect themselves. The ***NoCOV/HR*** cluster believed they were able to protect themselves from COVID-19 and that they were able to easily understand messages about protecting themselves.

**Conclusion:**

Understanding how PWUD conceptualize disease threat and using HBM can better inform interventions to improve future pandemic response. Findings suggest that resilience is key to protecting PWUD from future infectious disease outbreaks. Interventions aimed at increasing resiliency among PWUD may improve preventative behavior and decrease disease burden in this vulnerable population.

## Introduction

The COVID-19 pandemic caused significant disruptions to everyday life, specifically through “stay at home” orders and social distancing measures [1]. But vulnerable populations, such as people who use drugs (PWUD), were affected disproportionately due to significant social and environmental barriers that impeded their ability to act on COVID-related public health mitigation strategies, including unstable housing, the presence of chronic health conditions, poor access to bathrooms and running water, and having few financial resources to buy protective supplies such as masks, hand sanitizer, or other cleaning products [2–6]. Additionally, those using substances were more vulnerable to negative health consequences due to COVID-19. Tobacco or marijuana use can damage the respiratory system, opioid use increases risk of respiratory complications, and methamphetamine use cause increased lung pressure [7], all of which exacerbate the short- and long-term effects of COVID-19 and affect the overall health of the immune system. Those who use drugs may also not have been able to access correct risk communication messages provided by media or other communication channels, were hesitant to receive the vaccine, or did not prioritize their risk in the context of other daily risks as substance users, such as overdose and HIV or simply trying to find food and shelter [8].

The Health Belief Model (HBM) is used to understand failure of populations to adopt disease prevention strategies and proposes that a person’s belief in the threat of a particular disease along with their belief in the effectiveness of a proposed health-protective behavior predicts the likelihood that the person will perform the health-protective behavior [9, 10]. With six constructs—perceived susceptibility, perceived severity, perceived benefits, perceived barriers, cue to action, and self-efficacy—public health professionals have used this theory to help inform interventions to increase uptake of a particular behavior, like disease screening [9, 10]. The HBM has been used to understand and predict intention to receive the COVID-19 vaccine among several population groups, both in the United States and on a global health level [10, 11]. It has also been successfully applied to understand adherence to COVID-19 prevention practices [12–20], which are crucial to managing pandemics by encouraging public trust and safety [16]. An individual’s engagement in a preventative behavior can be encouraged via interventions that target HBM constructs [20]. In a cross-sectional survey study (n = 1,027) about COVID-19, HBM constructs including perceived benefits, perceived barriers, and cues to action were all significantly associated with the practice of preventative behaviors against COVID-19 [12]. Similarly, a study by Guidry et al. revealed that HBM constructs predicted uptake of most COVID-19 preventive actions, such as social distancing and washing hands, when controlling for demographics and psychosocial factors [15]. Al-Sabbagh et al. also found that perceived severity as well as benefits and barriers to preventative behaviors were significant predictors of adhering to quarantine regulations during the peak of COVID-19 [17]. Importantly, HBM may also clarify beliefs about COVID-19 and uptake of protective behaviors among PWUD, providing novel insights into the individual thought process that determine a person’s course of action to protect themselves against a future infectious disease pandemic.

Another construct outside of the HBM that is important to consider when trying to understand preventative behavior uptake for COVID-19 is resilience. Resilience—or the capacity to recover quickly from difficulties—can be a strong determinant of health, especially among vulnerable populations [21–23], such as PWUD. Resilience allows individuals and communities to prepare, respond to, and recover from difficulties—such as the COVID-19 pandemic [21, 23]. It is particularly important in disadvantaged communities and enhancing resilience is a major goal for many public health interventions among vulnerable populations [22]. By increasing resilience, it is possible to decrease population vulnerability to negative health events [22]. Simeon et al. found that higher levels of resilience among adults resulted in greater health outcomes compared to those with lower resilience, such as higher self-esteem, superior cognitive performance, and higher urinary cortisol levels [24]. Additionally, those who experience trauma, stigma, or other negatively impacting experiences tend to report low resilience and thus worse health outcomes [24]. Because PWUD often report trauma, stigma and negative experiences with healthcare professionals, it could be an especially important construct to measure and understand. In the context of COVID-19, an individual’s belief in their own ability to “bounce back” from a stressful event may impact uptake of preventative behaviors. But if they feel they cannot “bounce back,” they may also be less inclined to even try to protect themselves [23]. Drug use can impede overall resilience, especially so for people who have a more difficult time socializing with friends and family, are more fearful of COVID-19, and have overall poorer social and mental wellbeing [25]. We theorize that resilience may be associated with HBM constructs and self-reported engagement in COVID-19 mitigation strategies in PWUD, such that for individuals high in resilience, the relationship between HBM and self-reported engagement in mitigation is strengthened.

Thus, HBM and resilience can be used to better understand the decision-making process around protective behavior uptake, which is crucial for vulnerable populations such as PWUD during a pandemic. But the impact that COVID-19 has had on people with PWUD and what mitigation and protective practices were being used, is poorly understood. The aim of this study was to understand whether the HBM and its association with resilience could help explain decision-making about protective behavior uptake during the COVID-19 pandemic among PWUD to inform future interventions that enhance resilience and encourage proper use of preventative measures in a future disease outbreak in this vulnerable population [10].

## Materials and methods

These analyses utilized data from a mixed-methods study funded by the National Institute on Drug Abuse (1R34DA046305-03S). The study used qualitative (in-depth interviews) and quantitative (cross-sectional surveys) methods to explore beliefs about COVID-19, the perceived effects of COVID-19 on daily life and access to social services, vaccination beliefs, self-efficacy and resilience to carry out COVID-19 related protective behaviors among clients and staff of large harm reduction agency in Philadelphia, PA. The organization offers services to over 25,000 unique clients, including medication for opioid use disorder, behavioral health and infectious disease prevention, syringe exchange, medical treatment, and housing services.

A cross-sectional survey was developed based on qualitative findings. Research staff approached clients of the organization while they were receiving services and asked if they would be interested in taking a survey related to COVID-19. Those who were interested were taken to a private area to provide informed consent. Consented participants completed the survey with research staff on an iPad, in which research staff verbally administered the survey and entered responses into REDCap. Participants were provided with a paper survey and laminated scale sheet to improve visibility and comprehensibility. Surveys took approximately 15 min to complete and COVID-19 protocols, including mask wearing and social distancing, were maintained. Data collection occurred between September and December 2021. Participants received a $15 gift card upon completion. Temple University’s Institutional Review Board reviewed and approved this study (#27637).

### Participants

Eligible participants (n = 75) were: 1. Clients of the organization (i.e., accessing any of the services provided and had an ID number); 2. 18 years of age or over; and 3. Able to speak and read English.

### Measures

The survey instrument developed by the authors used both validated measures as well as study specific items based on findings from qualitative interviews with clients of the organization. It consisted of 18 sociodemographic questions and 22 sections related to COVID-19, vaccinations, harm reduction, sex work, drug use, and challenges to basic and community needs. All sections were presented in blocks of questions that corresponded to themes that emerged during prior qualitative analysis. Five sections corresponded specifically to HBM constructs. Items were expressed as statements and asked respondents to answer how much they agreed or disagreed with the statement on a zero (highly disagree) to 10 (highly agree) scale. These sections included:

• **Perceived Severity/Impact of COVID-19:** Items (n = 11) assessed different ways that COVID-19 impacted participants, such as through work opportunities, worsened living situations, increased mental health or sleep problems, increased use of alcohol or other substances, inability to access services, and increased verbal and physical conflict with others.
• **Perceived Susceptibility to COVID-19:** Items (n = 5) assessed risk of getting COVID-19, risk compared to others in the community, if they knew anyone who had gotten COVID-19, and if they changed their activities to prevent themselves from getting COVID-19.
• **Perceived Barriers to Protect from COVID-19:** Items (n = 8) assessed barriers to protecting oneself from COVID-19 such as difficulty following instructions, lacking patience to follow prevention instructions, using drugs with others making it difficult to socially distance, feeling uncomfortable wearing masks, or not having materials to use (mask, sanitizer).
• **Perceived Self-Efficacy to Protect from COVID-19:** Items (n = 7) assessed how confident and in control they felt in protecting themselves against COVID-19, how active they were to remain informed, and if they followed guidelines and took the steps to protect themselves.
• **Cues to Action (to Prevent COVID-19):** Items (n = 4) assessed whether they were reminded about safety tips against COVID-19 or needed to be reminded to do things to protect themselves, if news about how to protect themselves was accessible, and if messages about how to protect oneself from COVID-19 were easy to understand.

Other measures of interest included resiliency, which was measured with the 6 items of the validated Brief Resilience scale (answers on a 1 to 5 scale) [25], and 11 items on the impact of the COVID-19 Pandemic, based on Grasso et al’s work and modified for the population (answers yes/no) [26].

### Analytic plan

To examine associations between our constructs of interest, we performed a K-means cluster analysis [27]. Classification was based on four items aimed at assessing participants’ relationship with COVID-19: 1. If they have ever tested positive for COVID-19; 2. If they had known someone who had COVID-19; 3. If they believed they are susceptible of getting COVID-19 in the future; and, 4. A sum score of their perceived resiliency against difficult things. A non-hierarchical method is used in the K-means approach to clustering to discern latent subgroups within a sample. Individual cases are then assigned to a predetermined number of clusters according to their proximity to the nearest centroid (mean) of constituent items [27]. This is then performed iteratively until the desired number of clusters is produced. Considering the total sample size was 75 participants and there was missing data on 15 cases, the final sample size was n = 60. A two-cluster solution was specified and assessed for data fit. Once a cluster solution was found, the associations between membership in one of the two clusters and survey items from the five sections with HBM constructs were assessed using Student’s t-test to compare the means of continuous variables between the two clusters with an alpha value of 0.05 to determine statistical significance. Chi-squares were used to compare demographics and two binary HBM construct items. All analyses were done with SPSS v. 28.

## Results

### Cluster analysis

**FIGURE 1:**
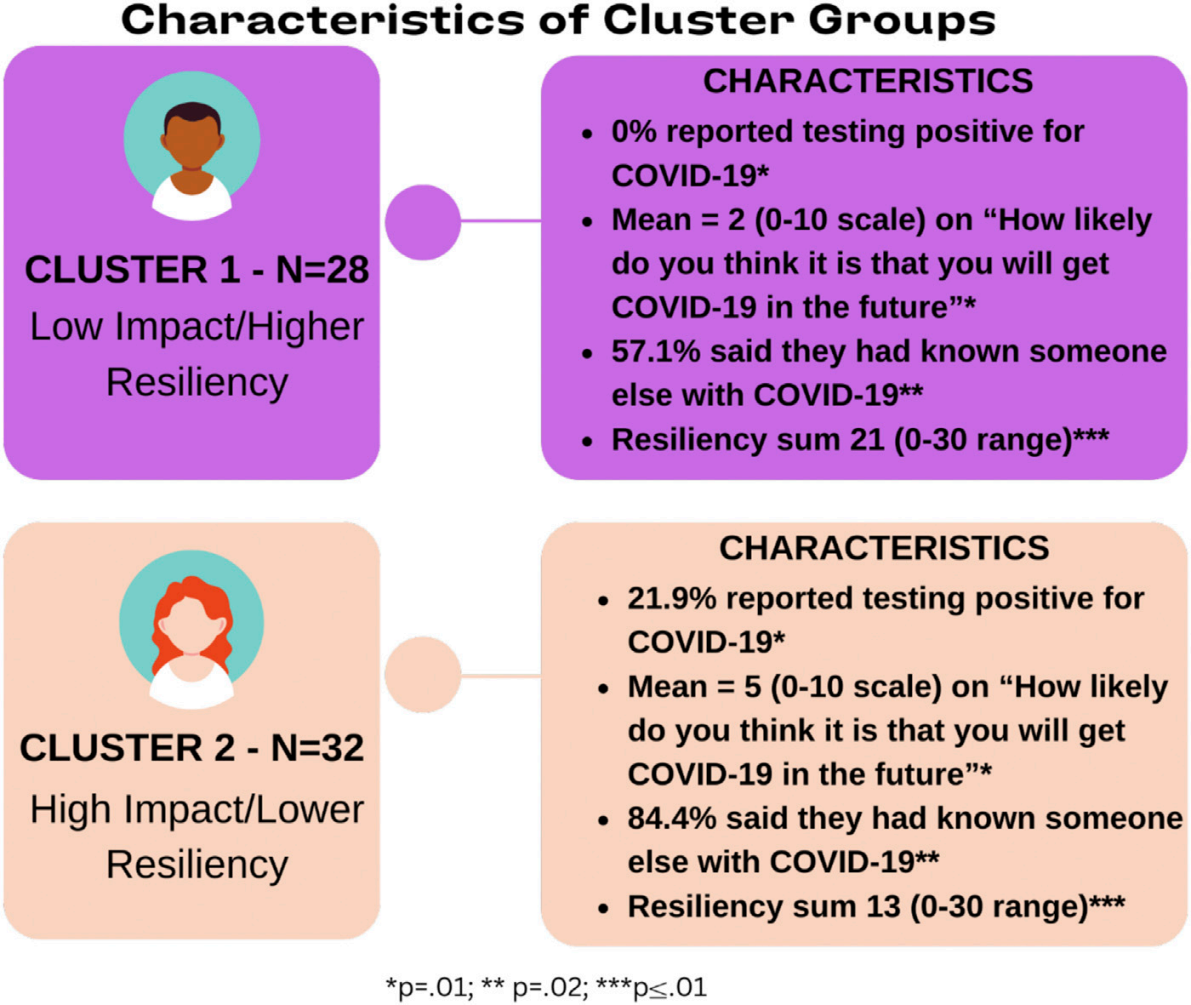
Differences between two groups on clustering variables.

**FIGURE 2:**
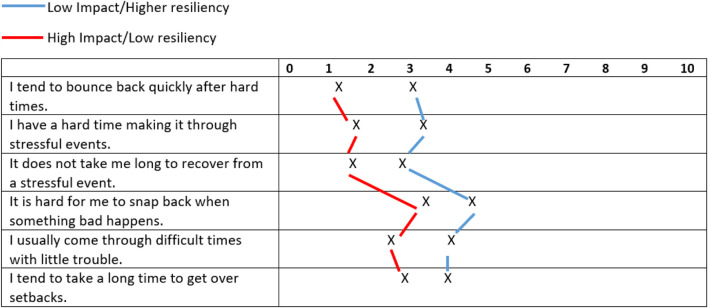
COVID-19 clusters—based on resilience.

Convergence in the cluster solution was achieved after 8 iterations. Differences between clusters based on their constituent items were analyzed to create definitions for both clusters. Figure 1 reports the means or percentages for each item delineated by cluster. Cluster 1 (n = 28, 47%) was more likely to believe that they had not been significantly affected by COVID-19 and that it was not a potential threat in the future (M = 2, *p* = 0.008), and had high overall resiliency during difficult times (M = 21, *p* = <0.01). Cluster 2 (n = 32, 53%) was more likely to believe that they had been significantly affected by COVID-19 or would be in the future (M = 5, *p* = 0.008), and had lower resiliency (M = 13, *p* = <0.01). We use the terms ***Less COVID impact/High resilience*** (***NoCOV/HR***) for Cluster 1 and ***High COVID impact/Low resilience*** (***COV/LR***) for Cluster 2 to apply common nomenclature to denote how segments differed by study variables. Figure 2 presents the two clusters with the means of their resiliency scores plotted to illustrate differences between the two groups.

### Sample demographics

Overall, most of the sample was white/Caucasian (61.6%), had finished high school or earned a GED (58.6%), was unemployed (75%), and male (60.3%). A little over half were COVID-19 vaccinated with at least one dose (56.7%) and most were knowledgeable about COVID-19 (mean score of 6.09 out of 7). Table 1 presents a summary of demographics by total sample and by cluster along with other variables of interest, including vaccination status, likelihood of being vaccinated if offered (among unvaccinated participants), and knowledge of COVID-19. No significant differences were observed between clusters on any demographic variables or other variables of interest.

**TABLE 1:** Sociodemographic variables of total analytic sample and by cluster.

	Total (n = 60)	Cluster 1 (n = 28) Low impact/Higher resiliency	Cluster 2 (n = 32) High impact/Lower resiliency	*p*
Race/Ethnicity				0.50
African American	11 (18.3%)	4 (14.8%)	7 (23.3%)	
Latino/a	6 (10.0%)	2 (7.4%)	4 (13.3%	
Native American	1 (1.6%)	1 (3.7%)	0 (0.0%)	
White/Caucasian	37 (61.6%)	18 (66.7%)	19 (63.3%)	
Multi-racial	1 (1.6%)	1 (3.7%)	0 (0.0%)	
Other	1 (1.6%)	1 (3.7%)	0 (0.0%)	
Educational Attainment				0.69
Less than high school	7 (12.1%)	4 (14.3%)	3 (10.0%)	
Finished high school or GED	34 (58.6%)	16 (57.1%)	18 (60.0%)	
Technical, vocational school, or community college	2 (3.4%)	0 (0.0%)	2 (6.7%)	
Some college	13 (22.4%)	7 (25.0%)	6 (20.0%)	
College degree or above	2 (3.4%)	1 (3.6%)	1 (3.3%)	
Employment Status				0.58
Employed full time	3 (5.4%	2 (7.1%)	1 (3.6%)	
Employed part time	9 (16.1%)	5 (17.9%)	4 (14.3%)	
Not working but looking for work	25 (44.6%)	11 (39.3%)	14 (50%)	
Not working and not looking for work	17 (30.4%)	8 (28.6%)	9 (32.1%)	
Other	2 (3.6%)	2 (7.1%)	0 (0.0%)	
Income Level (past month, from all sources)				0.06
$0-$500	12 (22.6%)	8 (29.6%)	4 (15.4%)	
$501-$1,000	22 (41.5%)	7 (25.9%)	15 (57.7%)	
$1,001-$2,000	11 (20.8%	9 (33.3%)	2 (7.7%)	
$2,001-$3,000	4 (7.5%)	2 (7.4%)	2 (7.7%)	
$3,001-$4,000	2 (3.8%)	0 (0.0%)	2 (7.7%)	
$4,000 or more	2 (3.8%	1 (3.7%)	1 (3.8%)	
Engaged in sex work during the pandemic (yes)	12 (20.0%)	7 (21.9%)	5 (17.9%	0.58
Homeless (within past 6 months)	31 (55.4%)	14 (50.0%)	17 (60.7%)	0.82
Sex Assigned at Birth				0.26
Male	35 (60.3%)	19 (67.9%)	16 (53.3%)	
Female	23 (39.7%)	9 (32.1%)	14 (46.7%)	
Gender Identity				0.24
Female	22 (37.9%)	8 (28.6%)	14 (46.7%)	
Male	35 (60.3%)	19 (67.9%)	16 (53.3%)	
Transgender Female	1 (1.7%)	1 (3.6%)	0 (0.0%)	
Vaccinated				0.65
No	26 (43.3%)	13 (46.4%)	13 (40.6%)	
Yes (at least one dose)	34 (56.7%)	15 (53.6%)	19 (59.4%)	
Likelihood of Being Vaccinated if Offered (0–10)	5.51 (4.00)	5.62 (4.19)	6.23 (4.23)	0.71
Knowledge about COVID-19 (sum score, 0–7)^a^	6.09 (1.01)	6.22 (1.05)	5.97 (0.98)	0.35

^a^ Calculated by summing up a score with each participant’s responses to 7 true false items. If they answered correctly, they would get a 1 for that item, incorrect a 0. The minimum score could be a 0 and maximum 7 for perfect knowledge.

### Perceptual variables

Means, standard deviations, t-scores, *p*-values, and Cohen’s d are reported in Table 2 for all survey items from the five HBM-related groups that required a Student’s t-test. Percentages, Pearson’s chi square, and *p*-values are reported in Table 3 for binary survey items that required a chi-square test. Significant results by section are described below and report only the significant items in which the two clusters differed (at the 0.05 significance level or below).

**TABLE 2:** Significant differences between clusters on HBM variables: T-test results.

	Cluster 1 (n = 28) Low impact/Higher resiliency	Cluster 2 (n = 32) High impact/Lower resiliency	*t*	*p*	Cohen’s *d*
People I know have gotten COVID-19. (0–10 scale)	5.61 (4.61)	8.09 (3.24)	2.43	0.02	3.96
It is difficult to follow instructions to prevent the disease (i.e., mask wearing, social distancing). (0–10 scale)	0.63 (1.90)	3.94 (4.02)	3.91	<0.001	3.23
I do not have things to keep me safe from COVID-19 (mask, hand sanitizer). (0–10 scale)	0.67 (1.82)	2.16 (3.22)	2.13	0.04	2.68
I know what is safe and not safe to protect myself from COVID-19. (0–10 scale)	9.29 (1.33)	8.38 (2.03)	2.03	0.05	1.74
Messages about how to protect myself and others from COVID-19 are easy for me to understand. (0–10 scale)	9.71 (0.85)	8.81 (1.91)	2.32	0.02	1.50

**TABLE 3:** Significant differences between clusters on HBM variables: Chi-square results.

	Cluster 1 (n = 28) Low impact/Higher resiliency	Cluster 2 (n = 32) High impact/Lower resiliency	χ^2^	*p*
Had to continue to work in close contact with people who might be infected. (% agree yes)	26.9%	58.1%	5.57	0.02
Increase in mental health problems or symptoms (e.g., mood, anxiety, stress). (% agree yes)	46.4%	71.9%	4.03	0.05

### Perceived severity/impact of COVID-19

There were two significant items between the two clusters. More people in the ***COV/LR*** cluster agreed that they “had to continue to work/be in close contact with people who might be infected” (28.9% vs. 58.1%, χ^2^ = 5.569 *p* = 0.018). Additionally, this cluster was more likely to agree that they had experienced an “increase in mental health problems or symptoms (e.g., mood, anxiety, stress)” during COVID-19 (46.4% vs. 71.9%, χ^2^ = 4.029, *p* = 0.045).

### Perceived susceptibility to COVID-19

The ***COV/LR*** cluster more strongly agreed with the statement “People I know have gotten COVID-19” relative to the ***NoCOV/HR*** cluster [M = 5.61, SD = 4.61 vs. M = 8.09, SD = 3.29; t (58) = 12.56, *p* = 0.02].

### Perceived barriers to protection from COVID-19

The ***COV/LR*** cluster was more likely to agree with the statement “it is difficult to follow instructions to prevent the disease (i.e., mask wearing, social distancing),” [M = 0.63, SD = 1.90 vs. M = 3.94, SD = 4.02; t (57) = 42.61, *p* = <0.0001s] and “I don’t have things to keep me safe from COVID-19 (mask, hand sanitizer),” [M = 0.67, SD = 1.82 vs. M = 2.16, SD 2.22; t (57) = 10.31, *p* = 0.04].

### Perceived self-efficacy to protect from COVID-19

One item was found to be significant. The ***NoCOV/HR*** cluster agreed more strongly with the statement “I know what is safe and not safe to protect myself from COVID-19,” [M = 9.29, SD = 1.24 vs. M = 8.38, SD = 2.02; t (58) = 4.57, *p* = 0.05].

### Cues to action (to prevent COVID-19)

One item in cues to action was significant, where the ***NoCOV/HR*** cluster agreed more strongly with the statement “messages about how to protect myself and others from COVID-19 are easy for me to understand” [M = 9.71, SD = 0.85 vs. M = 8.81, SD = 1.91; t (57) = 20.10, *p* = 0.02].

## Discussion

COVID-19 research has not previously identified how PWUD conceptualize disease on an intrapersonal level and the potential association with resiliency. This study identified two distinct cluster groups from a sample of clients of a large harm reduction organization which delineated people by their perceived personal impact of COVID-19 and their general resiliency during difficult situations. Importantly, these two clusters did not differ by demographic characteristics, indicating clusters were independent of age, gender, race or other potential descriptors that have previously been used to compare populations by COVID-19 behaviors [28, 29]. Instead, “psychographic” characteristics (i.e., perceptions or attitudes) defined the resulting clusters, a novel approach to understanding populations. Differences by disease conceptualization and perceived resiliency, a previously unexplored association in PWUD and COVID-19 research, were observed based on this approach.

This study also found relationships between these cluster groups and HBM constructs, suggesting that aside from other social and structural barriers that PWUD face, there are significant intrapersonal barriers that impede uptake of preventative behaviors. Conceptualizing participants by their perceived impact and resiliency and then identifying them by their perceived severity and susceptibility, perceived barriers and self-efficacy to protecting themselves, and cues to action to prevent COVID-19 could be helpful in thinking about future interventions during an ongoing pandemic, especially among vulnerable populations like PWUD [12–15, 17–20]. Those in the ***COV/LR*** cluster felt they were less able to follow instructions or keep themselves safe during COVID-19. They also indicated being less able to access preventative items, such as masks or hand sanitizer, highlighting a possible need for increasing education or communication, as well as providing easy access to these products to mitigate these perceived barriers. Clearly, even though the fear of COVID-19 impact was evident, those in this cluster were less confident in their ability to adhere to preventative behaviors, similar to the findings of Kamran et al. and Maunder [29, 30]. These differences could be helpful to inform community-based strategies on promoting protective behaviors during COVID-19 or other infectious disease outbreaks among a vulnerable population, such as PWUD. Importantly, regardless of the cluster, answers to HBM based items as well as overall resiliency indicates that respondents often fall on the same end of the disagree or agree scale, but they slightly diverge in terms of magnitude of agreement or disagreement. Thus, messaging or other types of interventions aimed at improving resiliency for everyone should result in an improvement among both groups. Findings can be used as a guide on how best to move individuals towards health protecting behavior uptake especially when they are presenting anxiety, uncertainty, apprehension, or skepticism of government information about prevention [18]. It is also crucial to understand how particular constructs interact with one another. For example, Carico et al. found that an individual is more likely to use preventive COVID-19 behaviors if they perceive the threat of the disease as large [18]. Interventions that can increase feelings of perceived severity and perceived susceptibility may also increase intervention impact and in turn affect preventive behaviors [18]. Acting upon these three constructs is theorized to potentially have the most powerful effect on improving uptake of preventive behaviors [18]. Further research on how these constructs interact with other important barriers in vulnerable populations, such as we found with introducing the concept of resilience or the significant structural barriers PWUD have in managing everyday needs, necessitates further investigation.

Notably, this study also highlighted the connection between the performance of preventative behaviors and resilience. Three resilience principles—managing connectivity, enhancing learning, and the management of feedback—were found to have shaped the initial response to the COVID-19 pandemic [31]. Similar to Berbés-Blázquez et al., we also found that perceived resilience drove individual response to COVID-19 through the enaction of preventative behaviors, as seen through the differences between the two clusters by each HBM construct [31]. The experience of the pandemic itself can also shape resilience, where those who perceived COVID-19 to be a high threat to themselves showed less resilience, a finding that was also found in Manchia et al.’s work [32]. The added negative effect of COVID-19 on the psychological wellbeing of PWUD, which is also directly correlated to reduced resilience and thus less efficacy in performing preventative behaviors, is an important association [31, 32]. We also found this to be true, where those who had lower resilience also reported an increase in mental health problems. Importantly, COVID-19 restrictions may have exacerbated these negative outcomes due to government-enforced curfews and decreased capacity of community agencies to provide life-protecting harm reduction services to PWUDs, leading to increased drug use and feelings of isolation and decreased resilience [33]. Our findings on resilience highlight the need for community interventions to strategize novel ways to increase resilience among PWUD, as this may better prepare them for protection from future disasters, such as another pandemic [34].

Notably, we did not find differences between clusters on any other non-HBM topic blocks such as knowledge of COVID-19, trust of information sources, coping, COVID-19 beliefs, COVID-19 vaccine beliefs, and neighborhood and community needs, highlighting how important the HBM constructs as well as resiliency are to consider (data not reported here). Thus, this study illustrates the stability of these two clusters, which implies that these constructs could be used at the community level to better inform practitioners who are working with PWUD in how best to address individual needs while also addressing larger structural barriers to preventive behavior. For example, easy screeners could be incorporated to assess overall resilience or beliefs in disease severity/susceptibility. This could enable staff to quickly assess where a client is in their thinking and tailor communication or intervention to them. Overall, an important piece of the puzzle would be to increase perceived threat of COVID-19 so that PWUD can act upon this threat. But increasing resiliency and perceived self-efficacy to use preventative behaviors is key. This may include targeting the susceptibility and severity constructs of the HBM, such as ensuring messages are catered to the way PWUD do or do not believe they are susceptible to an infectious disease, such as COVID-19, and then helping increase self-efficacy through cues to action or increasing access to what they need to protect themselves. Another piece of the puzzle is improving resiliency so that people who believe that COVID-19 poses a high threat to their health can also believe that if they do contract it, they can “bounce back” from it. This may include some community building techniques among PWUD to create a resilient community at large [35].

There are some limitations. Due to the nature of cross-sectional data, temporality inferences cannot be made. The survey also relies on self-reported data, so social desirability bias should also be considered. Additionally, results may not be generalizable to the broader PWUD population, as the study was conducted only in Philadelphia and among PWUD who are clients of a large social services agency that provides syringe exchange and other harm reduction services. This organization also adapted their operations in light of the pandemic, and these adaptations likely resulted in the sample feeling resourced and also feeling like they knew how to stay safe; this may not be the case had we sampled PWUD who do not have access to a large organization that provides services [8]. Those in other geographic areas may perceive the threat from COVID-19 differently or experience different levels of resilience. Additionally, our measures of threat and resilience may not fully capture their respective domains, thus limiting the content validity of the constructs we used for clustering. The relatively small sample size also limited the possibility of using alternate statistical models and/or controlling for covariates. Finally, we acknowledge that one of the clustering variables represented potential threat of COVID-19, which is very similar to the HBM construct of perceived susceptibility. Therefore, it makes sense that the item within perceived susceptibility was significant, as they are measuring similar things.

## Conclusion

Understanding how a vulnerable population, such as PWUD, conceptualizes disease threat and its association to overall resiliency to respond during the COVID-19 pandemic can better inform interventions to improve future pandemic response among PWUD. This study found relationships among HBM constructs, implying that aside from other social and structural barriers that PWUD face, there are significant intrapersonal barriers that impede uptake of preventative behaviors. Additionally, the significant findings in this study suggest that resilience is key to protecting PWUD from future infectious disease outbreaks. Interventions aimed at increasing resiliency among the PWUD community may be an important step to improving preventative behavior uptake and decreasing disease burden among this vulnerable population.

## Data Availability

The raw data supporting the conclusions of this article will be made available by the authors, without undue reservation.
